# The Florence Nightingale Effect: Organizational Identification Explains the Peculiar Link Between Others’ Suffering and Workplace Functioning in the Homelessness Sector

**DOI:** 10.3389/fpsyg.2016.00016

**Published:** 2016-01-28

**Authors:** Laura J. Ferris, Jolanda Jetten, Melissa Johnstone, Elise Girdham, Cameron Parsell, Zoe C. Walter

**Affiliations:** ^1^School of Psychology, University of Queensland, BrisbaneQLD, Australia; ^2^Institute for Social Science Research, University of Queensland, BrisbaneQLD, Australia

**Keywords:** social identity, social pain, vicarious trauma, burnout, job satisfaction, infrahumanization

## Abstract

Frontline employees in the helping professions often perform their duties against a difficult backdrop, including a complex client base and ongoing themes of crisis, suffering, and distress. These factors combine to create an environment in which workers are vulnerable to workplace stress and burnout. The present study tested two models to understand how frontline workers in the homelessness sector deal with the suffering of their clients. First, we examined whether relationships between suffering and workplace functioning (job satisfaction and burnout) would be mediated by organizational identification. Second, we examined whether emotional distance from clients (i.e., infrahumanization, measured as reduced attribution of secondary emotions) would predict improved workplace functioning (less burnout and greater job satisfaction), particularly when client contact is high. The study involved a mixed-methods design comprising interview (*N* = 26) and cross-sectional survey data (*N* = 60) with a sample of frontline staff working in the homelessness sector. Participants were asked to rate the level of client suffering and attribute emotions in a hypothetical client task, and to complete questionnaire measures of burnout, job satisfaction, and organizational identification. We found no relationships between secondary emotion attribution and burnout or satisfaction. Instead, we found that perceiving higher client suffering was linked with higher job satisfaction and lower burnout. Mediation analyses revealed a mediating role for identification, such that recognizing suffering predicted greater identification with the organization, which fully mediated the relationship between suffering and job satisfaction, and also between suffering and burnout. Qualitative analysis of interview data also resonated with this conceptualization. We introduce this novel finding as the ‘Florence Nightingale effect’. With this sample drawn from the homelessness sector, we provide preliminary evidence for the proposition that recognizing others’ suffering may serve to increase job satisfaction and reduce burnout – by galvanizing organizational identification.

## Introduction

In human services such as the homelessness sector, frontline workers are often faced with confronting circumstances in their daily work with clients. Operating within this environment can be demanding for workers, both professionally and personally (Baker et al., 2007). Their client base consists of individuals, couples, and families from diverse backgrounds who are at imminent risk or in the midst of housing crisis. People experiencing homelessness are stigmatized and often present with complex needs, and the transition into homelessness is marked by very high levels of psychological distress (Harris and Fiske, 2006, 2007; Fitzpatrick et al., 2013).

### Meeting Competing Demands: Care, Burnout, and Emotional Distance

A range of traumatic antecedents can catalyze entry into homelessness (Chigavazira et al., 2013), such as escape from domestic and family violence, or sexual and other forms of abuse; financial difficulties, unemployment, and poverty; family breakdown or bereavement; addiction or substance misuse; eviction or blacklisting from the private rental market; contact with the criminal justice system; mental illness; cultural conflict and intergenerational trauma; and many other triggers (Department of Families, Housing, Community Services and Indigenous Affairs, 2008; Australian Institute of Health Welfare, 2014).

Over and above the skills necessary to support people to cope with or exit homelessness, workers need the ability to remain resilient in the face of these challenges. Seeing clients’ pain and suffering can expose workers to vicarious emotional distress, with workers in the human services being particularly at risk (Maslach and Pines, 1977; Miller et al., 1995; Maslach et al., 2001; Baker et al., 2007; Bride, 2007; Gleichgerrcht and Decety, 2013).

Exposure to distressing human circumstances means workers may be vulnerable to workplace stress, and over time, these stresses can lead to *burnout*. Burnout is described as a ‘prolonged response to chronic emotional and interpersonal stressors on the job’, and is linked with a raft of negative consequences for the individual, their clients, and the broader workplace (Maslach et al., 2001; Maslach, 2003, p. 189). Burnout generally comprises three principle symptoms: exhaustion, perceived lack of accomplishment, and callousness (Maslach et al., 2001; Haslam and Reicher, 2006; Reicher and Haslam, 2006; Reicher et al., 2008). These symptoms are considered to emerge in response to specific workplace factors: accomplishment is undermined when people feel they do not have the resources to complete their tasks (such as time, training, or tools, and infrastructure), while exhaustion and callousness are associated with ongoing work overload and social stressors (Maslach et al., 2001). There is considerable evidence that burnout is linked with low job satisfaction (Lee and Ashforth, 1996).

Given these risks, it is important to understand how workers can avoid burnout and protect themselves emotionally from the difficulties and suffering of their clients, while at the same time providing those very clients support and care. We tested two models to better understand the implications of exposure to others’ suffering for frontline workers in this field. We examined whether workers in this field might protect themselves from the consequences of exposure to suffering and maintain positive workplace functioning via identification with the organization (i.e., a mediational hypothesis). We also examined whether emotional distance from clients would predict improved workplace functioning, particularly when client contact is high. (i.e., a moderation hypothesis).

### The Suffering of Others and Organizational Identification

The social identity approach proposes that a person’s group memberships and social categories dynamically inform one’s self-concept and position relative to other individuals and groups (Tajfel and Turner, 1979; Brewer, 1991; see also Hornsey, 2008, for review). In the workplace, an individual’s interrelatedness with the organization or organizational unit can be readily conceptualized in social identity terms (Ashforth and Mael, 1989; Haslam et al., 2003b; van Dick and Haslam, 2012). But how might acknowledging clients’ suffering promote identification with the organization, and how does this protect workers?

One possible explanation arises when considering how workers forge a positive identity in the workplace despite adverse aspects of the role. Exposure to clients who are suffering and working with people experiencing homelessness may be considered ‘dirty work’, because it involves contact with stigmatized members of society (Hughes, 1958; see also Ashforth and Kreiner, 1999; Ashforth et al., 2007; Baran et al., 2012). Such work can be considered noble or heroic – such as work done by firefighters, veterinarians in attending to animal euthanasia, surgeons, and carers for the elderly (Ashforth and Kreiner, 1999; Stacey, 2005; Baran et al., 2012). However, while those outside the profession may be grateful for the important work being done, they may also be pleased and relieved they do not have to carry it out themselves (Ashforth and Kreiner, 1999).

Importantly, Ashforth and Kreiner (1999) highlight the capacity for dirty tasks themselves to generate meaning, where negative aspects of the job create and maintain organizational identity, for instance by allowing workers to display resilience and fortitude or to demonstrate sacrifice in a way that carries collective significance. In particular, for workers in the homelessness sector, acknowledging clients’ suffering could fuel meaningfulness by creating an immediate and salient link between the work and its purpose – to *relieve* suffering. In this way we can conceptualize others’ suffering as a potential catalyst for organizational identification: theoretically, acknowledging suffering could enliven a sense of shared purpose and meaning in the workplace and enhance identification with the organization (Haslam et al., 2003a,b; van Dick and Haslam, 2012).

### Identification with the Organization and Workplace Functioning

There is a large literature on the benefits of group memberships, and identifying with the organization has been consistently linked with positive workplace outcomes. Shared social identity promotes communication (Greenaway et al., 2015), provides a basis for shared social capital (Cornelissen et al., 2007), predicts positive organizational citizen behaviour (Christ et al., 2003), and relationally binds groups to their leaders (Steffens et al., 2014). A raft of evidence shows the benefits of social identity and group memberships in terms of general wellbeing (Haslam et al., 2005, 2009; Haslam and Reicher, 2006; Iyer et al., 2009).

Relevantly, van Dick and Haslam (2012) point to empirical and meta-analytical work linking high organizational identification with higher job satisfaction, and lower levels of stress (see for example, Haslam et al., 2005; Riketta and Dick, 2005). They argue that the capacity for a workplace stressor to enliven stress is moderated by how relevant it is to salient organizational identities. This suggests that for high organizational identifiers, stressors which go to the heart of one’s organizational identity have the potential to be more damaging. However, van Dick and Haslam (2012) further point out that these identity salient stressors also create the conditions for collectively derived responses to shared problems (Haslam et al., 2005) and access to social support (Haslam et al., 2004). This suggests that organizational identification furnishes individuals with additional resources to deal with the challenges they face together, leading to more positive workplace outcomes. In sum, the social identity approach provides a strong and plausible explanation for how workers might marshal psychological resources to deal with their clients’ suffering, particularly in stigmatized or lower-status industries.

### Building a Protective Barrier Through Emotional Distancing

A growing literature on humanness and dehumanization indicates a possible alternative for how workers protect themselves from the emotional challenges of caring for others who are suffering. Empathy is associated with positive outcomes for care recipients in therapeutic contexts (see for example, Halpern, 2003; Haslam, 2007; Haque and Waytz, 2012). However, distancing oneself emotionally from challenging material might help to preserve those emotional resources that are tapped when extending empathic concern and perspective-taking in relation to clients. Schulman-Green (2003) reported qualitative evidence that health employees engage in emotional distancing as a coping mechanism, such as referring to patients in terms of their condition rather than by their names. In interviews with intensive care nurses, Cadge and Hammonds (2012) found that staff expressed concern for patients but also detailed efforts to maintain emotional barriers.

Recent quantitative evidence suggests that emotional distancing among health care workers is associated with improved coping with patients’ physical pain and mortality (Vaes and Muratore, 2013; Trifiletti et al., 2014). In a cross-sectional study with 78 Italian hospital and oncology unit workers, Vaes and Muratore (2013) found that workers who emotionally distanced themselves by reporting lower presence of uniquely human emotions (also termed ‘secondary emotions’) in a hypothetical patient tended to show more perceived professional efficacy, and more work engagement. Notably, the relationship between this form of emotional distancing and burnout was moderated by patient contact: for those health workers with high levels of patient contact, reporting higher presence of uniquely human emotions was associated with higher disillusionment, psycho-physical exhaustion and professional inefficacy. Trifiletti et al. (2014) reported similar findings in a study involving 109 nursing staff. They found that nurses’ self-reported stress was positively correlated with the attribution of uniquely human traits to patients; but only for those nurses with high overall affective commitment to patients and their organization. Reconciling these findings with Vaes and Muratore’s (2013) study, it appears that emotional distancing is linked with reduced burnout for health workers, especially those with high-contact roles, or those who are particularly emotionally committed to and involved in the organization.

This makes it important to tease apart the concept of emotional distancing in order to understand what protection it might afford. From the outset, it is important to note that in the context of patient and client care, the present work seeks to explore targeted and subtle ways of engendering emotional distance from a dehumanization and infrahumanization framework (see Haslam, 2014, for review). We do not seek to impute that extreme denial of humanness is occurring in this context, nor anticipate extreme forms of dehumanization that represent a failure to extend the moral concern normally afforded to other human beings (Goff et al., 2008; Harris and Fiske, 2011). Instead, we are seeking to examine protective emotional distance in a challenging work context by using the theoretical framework of infrahumanization.

Infrahumanization can be considered a subtle form of humanness denial that operates at intergroup (Leyens et al., 2001; Demoulin et al., 2004b) and interpersonal levels (Bastian et al., 2014a; Haslam, 2014). This framework proposes that there are secondary or *uniquely human emotions*, and that these are different to the primary or basic emotions experienced by both humans and animals alike (*non-uniquely human emotions*). For example, primary emotions such as joy, pain, or fear do not distinguish an entity as being human, as animals too undergo these emotions. In contrast, secondary emotions are unique to humans, such as optimism, shame, or indignation, as these are emotions that animals are not considered to experience (Demoulin et al., 2004a). Ascribing secondary (uniquely human) emotions to an entity is therefore an index of infrahumanization, where lower attribution shows greater infrahumanization. Secondary emotion attribution represents a way to capture the concept of emotional distancing, in the sense that denial or suppression of secondary emotion characteristics might provide more direct information about how *emotional* distancing operates.

Moreover, the current evidence base on protective infrahumanization has only examined exposure to others’ physical pain and suffering. It remains an empirical question whether similar effects are observed when considering social pain. Modern approaches to homelessness conceptualize the experience and existence of homelessness as a symptom and expression of social exclusion (Minnery and Greenhalgh, 2007). Social exclusion may be considered as a form of *social pain*, which MacDonald and Leary (2005, p. 202) describe as ‘…a specific emotional reaction to the perception that one is being excluded from desired relationships or being devalued by desired relationship partners or groups’. Whether protective infrahumanization is observed on exposure to others’ social pain awaits empirical substantiation.

### The Present Research

Working in the homelessness sector is a challenging undertaking, and leaves workers exposed to the risk of burnout. We examined two models concerning the implications of exposure to clients’ suffering. First, building on the literature on social identity and dirty work, we proposed that acknowledging suffering can lead to the development of a positive organizational identity and this can protect workers in stigmatized roles by fostering meaningfulness. Second, an alternative literature points to emotional distancing as providing protection for workers exposed to the suffering of others. This proposes that ‘dialing down’ empathy and increasing emotional distance through infrahumanization is protective when empathetic interpersonal skills come under heavy demand – even though empathy is a key skill generally associated with positive client outcomes. While the existing evidence base has largely focused upon exposure to others’ physical pain (Vaes and Muratore, 2013; Trifiletti et al., 2014), we probed whether there is evidence for protective organizational identity or infrahumanization associated with exposure to others’ social pain. Accordingly, we aimed to examine whether infrahumanization is protective for staff working in homelessness service delivery, especially those with high client contact – with a view to examining links between infrahumanization and reduced burnout, and higher job satisfaction.

Thus the present study extends on previous literature by examining these two possibilities in a novel caregiving context: provision of support services to people experiencing homelessness. To that end, we combined qualitative and quantitative approaches. We interviewed and surveyed a sample of frontline homelessness service providers to discover their experiences and to investigate what factors contribute to job satisfaction and burnout.

## Materials and Methods

### Participants, Design, and Procedure

Ethical clearance was received from the University of Queensland Behavioral and Social Sciences Ethical Review Committee and the School of Psychology Ethics Review Committee, and gatekeeper approval for the study was secured from the employing organization. The sample consisted of 60 frontline service providers (18 male) between 23 and 65 years (*M*_age_ = 40.53 years) employed in homelessness service delivery roles. Staff members were considered in-scope if their employment duties included case management of homeless clients, outreach services, and/or general support duties involving direct contact with clients (collectively termed ‘frontline’ duties).

We employed a mixed-methods design comprising qualitative (interview) and quantitative (survey) components. The qualitative component explored workers’ experiences in direct service provision with clients, while the quantitative component was cross-sectional in design and measured client contact, infrahumanization, and client suffering (case history task), burnout, job satisfaction (workplace functioning), and organizational identification. We also took demographic and basic workplace information such as length of tenure.

**FIGURE 1:**
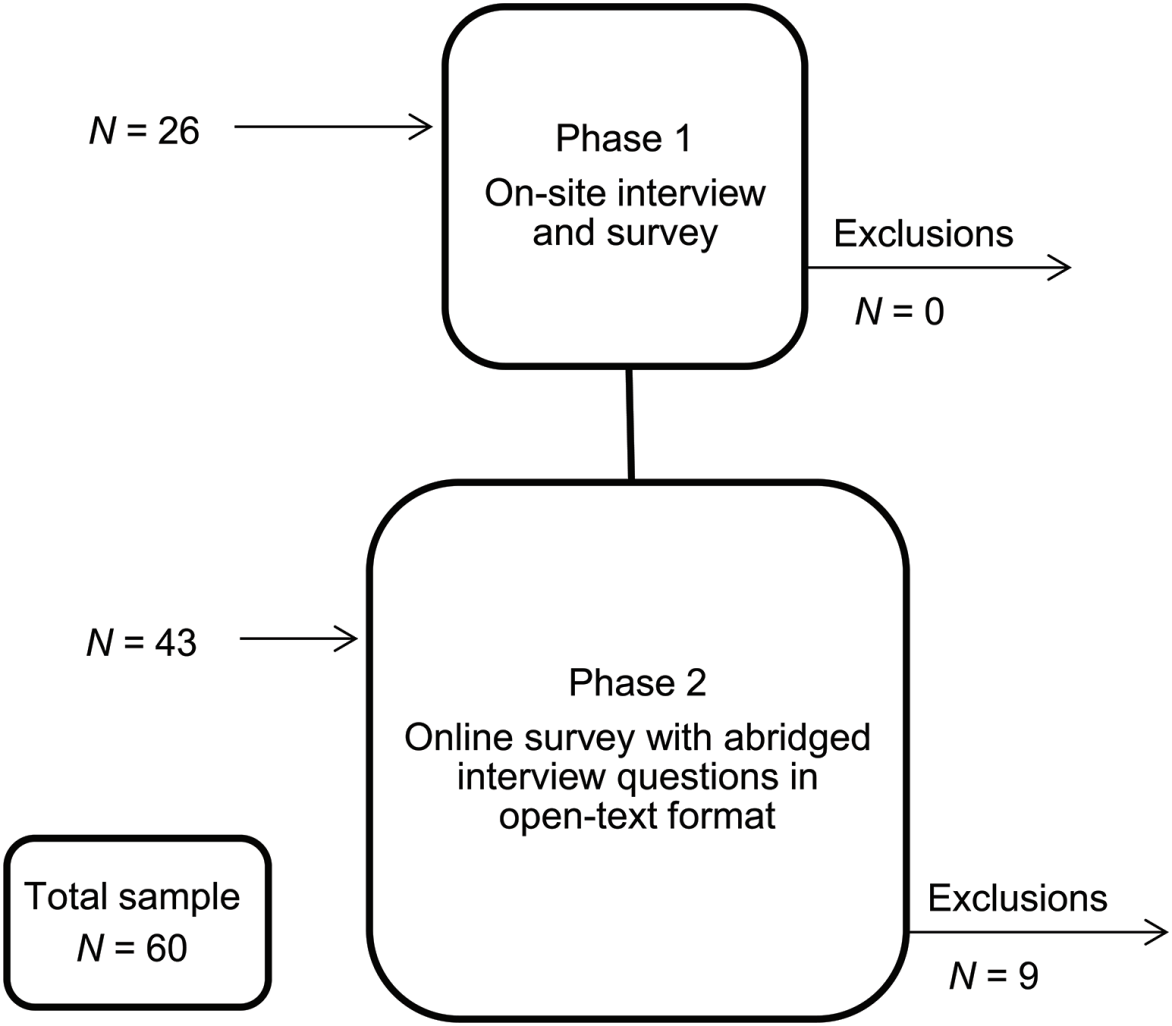
**Study recruitment flowchart for on-site and online phases, including sample size, and exclusions**.

Participants were first recruited for one-on-one semi-structured interviews and questionnaires delivered at the workplace (‘on-site phase’, see further below; *N* = 26). Recruitment was then extended to an online phase (*N* = 43) to ensure adequate sample capture, from which nine online participants who did not complete measures beyond initial demographic information were excluded. Overall, we aimed for a total sample of 60 participants for survey data across both recruitment phases, and closed data collection when the threshold of 60 completed responses was achieved (see **Figure 1**).

#### On-Site Phase

Interviews were carried out on-site to minimize disruption to service delivery, and ranged between approximately 15–60 min in duration (*M* = 31:41 min). On arrival participants were given study information, invited to provide informed written consent, and allocated a unique anonymous identifier to link interview and survey responses. Participants took part in the interview then completed the survey comprising demographics, client contact, case history task, workplace functioning, and organizational identification items. Finally, participants were verbally debriefed and thanked for their time.

#### Online Phase

Online participants were invited to visit the survey website at their convenience any time before the survey deadline. Once on the survey website, participants were given study information and invited to give informed consent by clicking a link to proceed. The online survey was presented in the same order as the on-site phase with the addition of four abridged interview questions at the end on empathy and self-care, adapted for an online open-text response format.

### Materials and Measures

#### Interview

The complete semi-structured interview comprised 22 questions on a series of topics relating to work role, motivation, belief systems, client outcomes, and factors contributing to or detracting from clients’ ability to exit homelessness. Specific to the present work were five questions tapping empathy (*To what extent do you empathize with clients and their circumstances?, How useful is empathizing with clients in helping them achieve positive outcomes?*), emotional connection with clients (*To what extent do you connect emotionally with clients?*), and questions on dealing with challenging experiences in the workplace and self-care (*How do you deal with challenging or confronting experiences in your role?, What sorts of self-care do you undertake, if any, to deal with difficult experiences in your role?*). The semi-structured interview format enabled participants to discuss their thoughts, feelings and experiences related to the interview topics. The interview was digitally recorded and transcribed for analysis. A shortened version of the interview questions was used during the online phase with four key questions relating to empathy, dealing with challenging experiences in the workplace, and self-care.

#### Survey

For both on-site and online phases, the survey consisted of the case history task, workplace functioning questionnaire, and demographic and basic workplace information items.

##### Case history task

**FIGURE 2:**
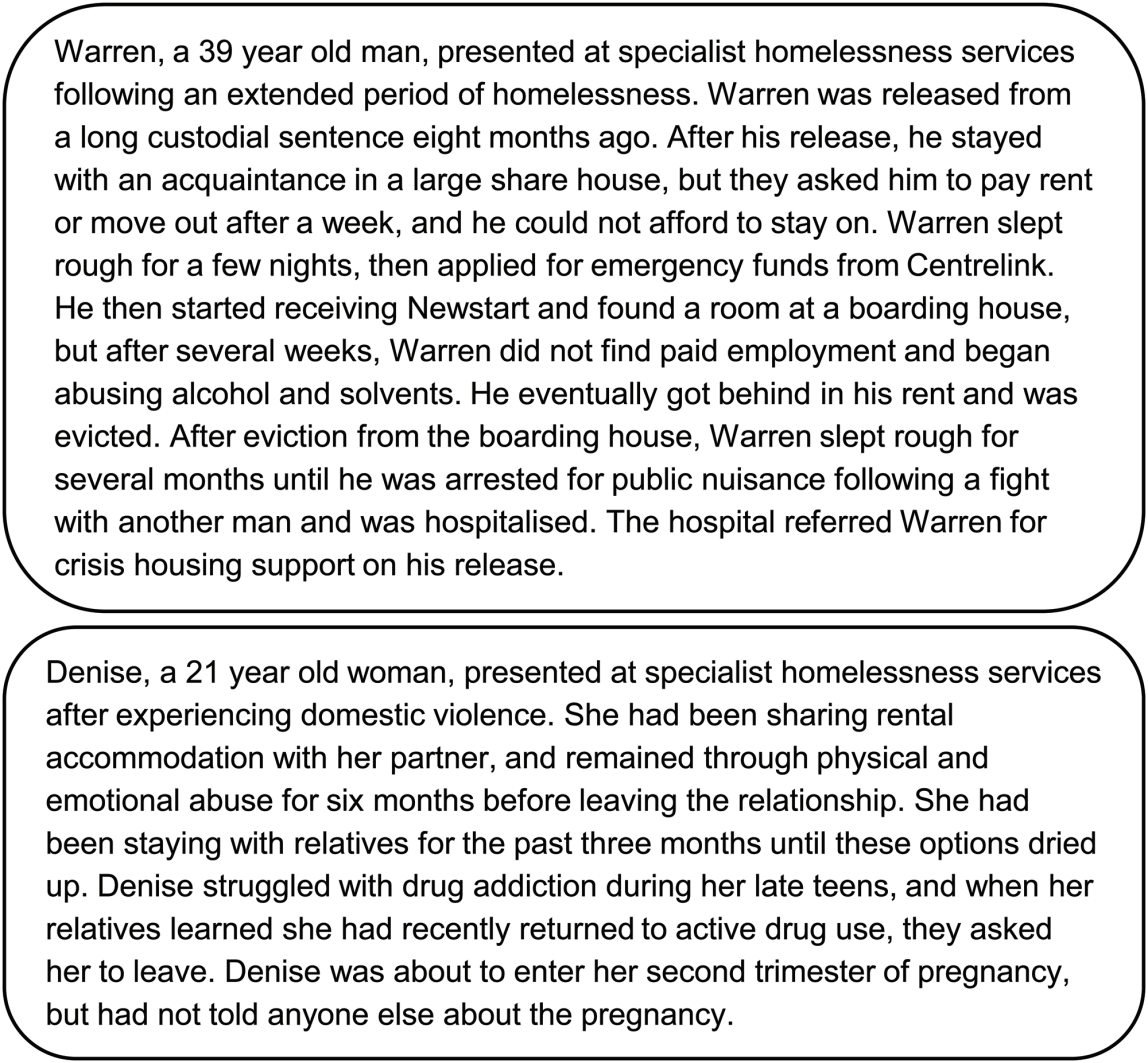
**Case history vignettes describing two hypothetical clients experiencing homelessness, ‘Warren’ and ‘Denise’.** ‘Centrelink’ and ‘Newstart’ are terms specific to the Australian national welfare system.

We developed two case history vignettes describing ‘Warren’, a 39 years-old man experiencing homelessness following a period of incarceration; and ‘Denise’, a 21 year-old woman escaping domestic violence. These vignettes were based on Vaes and Muratore’s (2013, p. 183) oncology patient “BM”, adapted to a homelessness context on the basis of national homelessness intake protocols and common client presentations drawn from pre-existing client interview data. Vignettes allowed us to measure participants’ responses to an individual client, rather than to ‘clients generally’, without breaching confidentiality obligations. Each vignette described the person’s circumstances using profession-relevant language without specifically referring to their emotional state (see **Figure 2**). In line with Vaes and Muratore’s (2013) analyses, we totalled the number of negative primary and secondary emotions attributed within each vignette. Measures between vignettes were moderately to highly correlated (ρs 0.44 to 0.86, *p*s < 0.01) with the exception of how often such a client was encountered^1^. Accordingly, we collapsed values over the vignettes to create a total value for negative secondary emotions and mean value for suffering in subsequent analyses^2^.

*Perceived client suffering and infrahumanization.* To measure perceptions of suffering, we asked participants to read the two hypothetical client case histories and rate client suffering (*How much is this client suffering?*) on a 7-point scale (from *Not at all* to *Extremely*). To measure infrahumanization, we asked participants to attribute emotions to the client described in each vignette. Participants were asked to indicate which (if any) emotions best described the client’s emotional state. Emotion options were largely negative in valence and included equal numbers of primary and secondary emotions (Vaes and Muratore, 2013). All 28 emotions were randomized and presented after each vignette. We gaged the extent to which participants attributed primary (non-uniquely human) and secondary (uniquely human) emotions when considering a client’s emotional state, and arrived at a total value for negative secondary emotions averaged across vignettes with lower scores indicating infrahumanization.

*Ancillary measures.* Participants were asked rate to what extent working with such a client would be *challenging, confronting*, or *distressing* on a 7-point scale (from *Not at all* to *Extremely*). We also asked participants to indicate on a 7-point scale (from *Never* to *Almost always*) how often they encounter such a client in their work situation. These measures aimed to respond to service delivery in the homelessness sector which is often divided by gender (Australian Institute of Health Welfare, 2014), such that a participant may deal exclusively with men or women depending on the center in which they work.

##### Workplace functioning and organizational identification

*Burnout.* We used the extended version of Haslam and Reicher’s (2006) burnout scale to quantify levels of workplace burnout in our sample (Jetten et al., 2012; see also Reicher and Haslam, 2006). This measure comprises three subscales: exhaustion, lack of accomplishment, and callousness. Each subscale is carried by three items, which participants rated on a 7-point scale (from *Do not agree at all* to *Agree completely*): exhaustion (*I feel I am working too hard at work, I feel energetic at work* (reversed), *I feel exhausted at work*; α = 0.60), lack of accomplishment (*At work I feel I am failing to achieve my goals, At work I feel frustrated, At work I feel I am accomplishing many worthwhile things* (reversed), α = 0.63), and callousness (*At work I am concerned about the welfare of others* (reversed), *At work I don’t really care what happens to people any more, At work I feel I am becoming callous toward other people*, α = 0.37). We noted the poor reliability of the callousness subscale, and further investigation revealed that this was attributable to one item (*At work I am concerned about the welfare of others*). The reliability^3^ of this subscale improves once this item is omitted (*r* = 0.68). This burnout scale also serves as a cohesive single measure of burnout by collapsing across the subscales (Jetten et al., 2012). Omitting the aforementioned problematic item from the callousness subscale improved the reliability of the overall burnout measure (α = 0.70), and this was used in subsequent analyses.

*Job satisfaction and organizational identification.* These variables were each measured with items on a 7-point scale (from *Do not agree at all* to *Agree completely*). We measured job satisfaction with three items (*All in all I am satisfied with my job, In general I don’t like my job* (reversed), *In general I like working here*, α = 0.74), which constitute the satisfaction subscale of the Michigan Organizational Assessment Questionnaire (Cammann et al., 1979; Jetten et al., 2012; van Dick and Haslam, 2012). We gaged organizational identification with two items (*I identify with this center, I identify with [the organization], r* = 0.63) designed for the specific requirements of this study (Postmes et al., 2013). We also measured demographics, including tenure in the homelessness sector (sector), tenure at the center location (center), and tenure in the present role or position (role).

## Results

### Quantitative Analyses

#### Suffering, Workplace Functioning, and Identification

Zero-order correlations^4^ between suffering, job satisfaction, and burnout (see **Table 1**) revealed that suffering was negatively correlated with burnout (*r* = -0.28, *p* = 0.029), and positively correlated with job satisfaction (*r* = 0.27, *p* = 0.038). **Table 2** shows correlations between length of tenure and key variables of interest.

**Table 1:** Descriptive statistics and zero-order correlations between perceived client suffering and workplace functioning variables.

Variable	*M (SD)*	1	2	3	4	5	6	7	8
(1) Suffering	6.32 (0.73)								
(2) Job satisfaction	5.96 (1.11)	**0.27**							
(3) Identification	5.12 (1.47)	**0.26**	**0.68^∗∗^**						
(4) Burnout	2.60 (0.81)	-**0.28**	-**0.58^∗∗^**	-**0.58^∗∗^**					
(5) Burnout (exhaustion)	3.10 (1.14)	-0.11	-**0.29**	-**0.30**	**0.79^∗∗^**				
(6) Burnout (lack of accomplishment)	2.79 (1.12)	-0.21	-**0.56^∗∗^**	-**0.51^∗∗^**	**0.82^∗∗^**	**0.38^∗^**			
(7) Burnout (callousness)	1.58 (0.81)	-**0.41^∗^**	-**0.57^∗∗^**	-**0.57^∗∗^**	**0.56^∗∗^**	0.20	**0.34^∗∗^**		
(8) Secondary emotions (negative)	8.15 (5.17)	0.07	-0.09	0.19	-0.01	-0.05	0.12	-0.11	
(9) Primary emotions (negative)	9.02 (5.26)	0.23	-0.02	0.21	0.00	0.01	0.08	-0.15	**0.79^∗∗^**

*N = 60. Text in bold indicates Pearson’s *r* is significant at *p* < 0.05; ^∗^*p* < 0.01; ^∗∗^*p* < 0.001 (two-tailed).*

**Table 2:** Descriptive statistics and zero-order correlations between length of tenure and workplace functioning variables.

	Length of tenure (years)		
	Sector	Center	Role
*Mean (SD)*	5.95 (6.44)	4.29 (4.99)	2.76 (2.84)
*Median*	3.42	3.00	2.00
*Minimum* (months)	1	1	1
*Maximum*	28	28	15
Suffering	-0.09	-0.06	-0.13
Job satisfaction	-0.15	-0.13	-0.13
Identification	-0.04	-0.09	-0.19
Burnout	0.24	0.23	0.24
Secondary emotions (negative)	**0.28**	0.18	0.04
Primary emotions (negative)	0.15	0.15	-0.05

*N = 60. Text in bold indicates Pearson’s *r* is significant at *p* < 0.05 (two-tailed). Tenure variables were positively skewed; correlations (but not descriptives) have been calculated following log transformation.*

**FIGURE 3:**
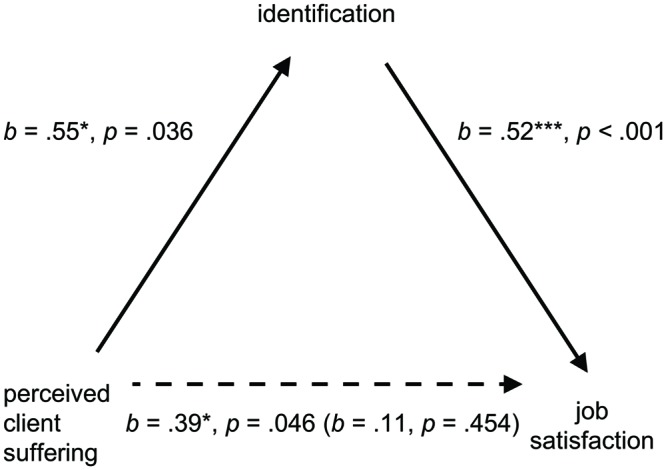
**Mediation model showing the effect of organizational identification on the relationship between perceived client suffering and job satisfaction.** Unstandardized coefficient in brackets relates to the direct effect once the indirect effect is accounted for.

We undertook mediation analyses^5^ to examine identification as a potential mediator, as a way to examine whether this might underlie the observed relationships between suffering and job satisfaction, and suffering and burnout (Baron and Kenny, 1986). First, we tested a bootstrapped mediation model with the PROCESS macro (Preacher and Hayes, 2008; Hayes, 2013) using 5,000 resamples in which suffering served as predictor, job satisfaction as outcome, and organizational identification as mediator. This provided evidence of full mediation, such that once the indirect effect of suffering via identification was accounted for [Indirect effect (*IE*) = 0.28, *SE* = 0.13, 95% Confidence interval (CI) = (0.08, 0.62)], the direct effect of suffering on satisfaction was no longer significant; see **Figure 3** for mediation model and coefficients).

**FIGURE 4:**
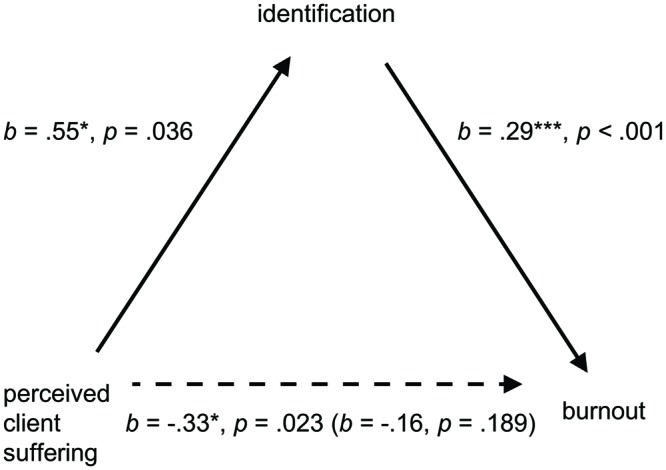
**Mediation model showing the effect of organizational identification on the relationship between perceived client suffering and burnout.** Unstandardized coefficient in brackets relates to the direct effect after accounting for the indirect effect.

Second, we tested a bootstrapped mediation model (Preacher and Hayes, 2008) using 5,000 resamples in which suffering served as predictor, overall burnout as outcome, and organizational identification as mediator. We again found evidence of full mediation, such that once the indirect effect of suffering via identification was factored in, the direct effect of suffering on burnout was no longer significant (*IE* = -0.16, *SE* = 0.07, 95% CI = [-0.34, -0.04]; see **Figure 4** for mediation model and coefficients). For completeness we also tested this at the subscale level, using three separate mediation models to test each burnout subscale as outcome variable. The indirect effect of suffering via identification was consistently evident for each of the burnout subscales (see **Table 3**).

**Table 3:** Additional mediation analyses examining the indirect effect of suffering on each burnout subscale via organizational identification.

Burnout subscale	*IE*	*SE*	*LLCI*	*ULCI*
Exhaustion	-0.12	0.21	-0.3411	-0.0049
Lack of accomplishment	-0.20	0.10	-0.4649	-0.0535
Callousness	-0.16	0.09	-0.4259	-0.0418

We also sought to test whether reverse mediation models could be supported by the data given the cross-sectional design we deployed. Specifically, this involved testing two models in which identification was retained as mediator, but where suffering served as the outcome variable, and satisfaction and burnout each as a predictor. First, with job satisfaction as predictor, we did not find support for mediation, with the indirect effect of job satisfaction via identification failing to account for a significant amount of the variance in suffering (*IE* = 0.08, *SE* = 0.08, 95% CI = [-0.08, 0.23]). Second, with burnout as predictor, we again failed to find support for mediation, with the indirect effect of burnout via identification unable to account for a significant amount of the variance in suffering [*IE* = -0.08, *SE* = 0.08, 95% CI = (-0.24, 0.06)].

#### Infrahumanization, Contact, and Workplace Functioning

We tested a moderation model using multiple regression with the PROCESS macro (Hayes, 2013), in which negative secondary emotion attribution (lower scores indicating infrahumanization) served as predictor, burnout as outcome, and client contact as moderator, measured by the rating scale item from Vaes and Muratore (2013). Variables were mean-centered for moderation via syntax for the PROCESS macro. We found no significant main effect of secondary emotion attribution on burnout (*b* = 0.00, *SE* = 0.02, *t* = 0.00, 95% CI = [-0.04, 0.04]), nor of contact on burnout [*b =* -0.16, *SE* = 0.14, *t* = -1.20, 95% CI = (-0.44, 0.11)], and no significant interaction [*b* = 0.02, *SE* = 0.03, *t* = 0.68, 95% CI = (-0.04, 0.07)]. Indeed, further moderated multiple regression analyses revealed no significant relationships between secondary emotion attribution and any of the three burnout subscales (exhaustion, lack of accomplishment, and callousness), nor any significant main effects or interactions arising from the introduction of any of the three indices of client contact as moderator, and we also failed to find evidence for these relationships using job satisfaction as the outcome variable (all *ns*, see **Table 4**). Noting that primary emotion attribution was also a poor predictor of workplace functioning variables (see **Table 1**), the pattern of results did not shift with the addition of primary emotion attribution or total emotion attribution as a covariate into the model.

**Table 4:** Multiple regression analyses of infrahumanization and workplace functioning measures, with client contact as moderator.

Outcome	Predictor	Coefficient	*SE*	*t*	*p*	*LLCI*	*ULCI*
Burnout	Secondary emotion attribution (infra)	0.00	0.02	0.00	0.996	-0.043	0.042
	Client contact %	-0.16	0.14	-1.20	0.235	-0.437	0.109
	Infra x contact	0.02	0.03	0.68	0.500	-0.036	0.073
Exhaustion subscale	Secondary emotion attribution (infra)	-0.02	0.03	-0.58	0.567	-0.079	0.044
	Client contact %	-0.12	0.19	-0.62	0.536	-0.509	0.268
	Infra x contact	-0.02	0.04	-0.55	0.583	-0.099	0.057
Callousness subscale	Secondary emotion attribution (infra)	-0.02	0.02	-0.97	0.336	-0.705	0.024
	Client contact %	-0.22	0.15	-1.47	0.148	-0.523	0.081
	Infra × contact	0.01	0.03	0.30	0.763	-0.052	0.069
Lack accomplishment subscale	Secondary emotion attribution (infra)	0.03	0.03	1.12	0.267	-0.025	0.139
	Client contact %	-0.17	0.19	-0.91	0.368	-0.539	0.203
	Infra × contact	0.06	0.04	1.75	0.086	-0.009	0.139
Satisfaction	Secondary emotion attribution (infra)	-0.01	0.03	-0.20	0.839	-0.064	0.052
	Client contact %	0.31	0.19	1.67	0.101	-0.062	0.679
	Infra × contact %	-0.03	0.04	-0.80	0.427	-0.104	0.045

### Qualitative Analyses

We also analyzed the qualitative data arising from interviews and the online open-text responses from frontline staff (Ritchie et al., 2014; Patton, 2015). We identified two cases with missing data for the qualitative component arising from the online phase, leaving *N* = 58 for qualitative analysis. While valuing the contribution of unique voices in the study (Kitto et al., 2008), owing to the small number missing the potential impact on qualitative analyses was considered to be tolerable.

These data were analyzed thematically with a top–down theoretical approach (Braun and Clarke, 2006), where themes were identified and analyzed which represented some level of patterned response or meaning from the interviews. We explored links between themes of suffering, organizational identification, and workplace functioning, in order to determine whether the relationships in our mediation models resonated with the lived experiences of our participants. We also examined the theme of empathy in workers’ practice with their clients, in order to better understand why links between infrahumanization and workplace functioning were not found in the quantitative data. Evidence of thematic references linking suffering, identification and/or workplace functioning are discussed below first, followed by themes of bounded empathy.

#### The ‘Florence Nightingale’ Effect

We implemented a theory-driven top–down analysis (Braun and Clarke, 2006) to unpack themes around acknowledging clients’ suffering, and whether this might be creating meaning, galvanizing organizational identification and thereby promoting improvements in workplace functioning. In support of findings of the mediation models from the quantitative data, we found links between themes of suffering, organizational identification, satisfaction, and burnout.

##### Suffering is why we are here

Workers recounted the difficulties they experienced in coming to terms with the pain and suffering their clients were feeling. However, acknowledging suffering was seen as an important step toward alleviating suffering.

[E]very person who you come across will have experienced trauma and we might not be able to relate to that trauma but being able to have a framework, to know how that can affect a person, is really important.

– Josie^6^

Workers also indicated that alleviating suffering motivated them to keep going in their role, and that this desire to lessen clients’ pain united them with the organization more broadly. Specifically taking action aimed at reducing clients’ pain was also a way to cope with the emotional after-effects of exposure to suffering and horror.

[Y]ou see some really broken women come through [the service] and their pain is bigger than them.

…

The clients [motivate me], definitely each and every one of them is unique in their own wonderful way. Just wanting to see them move forward, find stability.

– Sarah

##### Hard work is meaningful work

Workers reported deriving a fundamental sense of meaning and purpose from their role, despite – or even because of – its challenging nature. Some workers recounted actively seeking out more challenging work, and a preference for their current role over other types of work seen as easier but less meaningful.

I’ll be truthful with you – I really love the work. Now I know that sounds like one of those martyr statements, and I always cringe when I hear people say it, but I really love the work.

– Lucy

It was a change of life. I was in retail…and that wasn’t really giving me much fulfillment, I enjoyed it but I wasn’t really meaningful, so that’s when I went for this type of work…I mean it’s a lot…a lot harder as far as mentally and everything else …but this certainly is more fulfilling, more rewarding, helping people.

– Cath

[W]e’re not working on a puzzle or a jigsaw or a video game or something… you are connected and emotions are so raw and pure as well. A lot of conversations come around those emotions, a lot of great work comes around with someone telling you about exactly how they’re feeling.

– Dale

##### We are in this together

Some workers expressed a shared sense of solidarity in terms of their motivations and in facing workplace difficulties together, and that this helped them to function in their role.

[The job]…is not always easy because some things will click with you more than others and trigger you a little bit more but I guess that’s why you’ve got to have those steps in place, of supervision, and know your limits. I guess it’s the open communication with the team and letting them know. There’s [sic] been situations where I [felt]…, “I cannot deal with this situation.” – if it’s too close to home, and someone else steps up, and that’s just how you’ve got to work.

– Donna

For some, individual struggles and sacrifices in the workplace were reframed and contextualized within the collective; critically, these hardships offered deeper meaning and greater purpose when understood in the collective context.

It’s done with love, it’s the perfect fit…one team, one fight.

– Harry

I’ve seen a lot of burnout and I’ve had it myself, I’ve just had to learn how to work with that, because I love this industry.

– Dale

I think a big part for me is… the mission of the organization, I am aligned with that mission and that’s why I’m working for the organization, so I think it’s very broad in that sense the mission is to serve suffering humanity and I think a lot of suffering comes when people are homeless… and that’s where I feel like I’m aligned… and all the nitty gritty happens later but I think that’s where I’m aligned – that if this is the mission of the organization, this is what my mission is – to be an instrument in that big process of what it is to serve suffering humanity…

– Nadine

#### Bounded Empathy

We also examined the theme of empathy in workers’ practice with their clients. We again implemented a top–down theoretical approach to explore workers’ perspectives and experiences regarding empathy, and with an eye to better understanding why links between infrahumanization and workplace functioning did not emerge from the quantitative data. Responses were coded for presence or absence of reference to bounded empathy, conceptualized as any reference to the need for empathy, understanding, or authentic connection with clients, with the qualification that boundaries or limits were required. Of the 58 participants retained for qualitative data, 64% responses made specific reference to bounded empathy concepts.

##### Being strong and staying intact

An emerging theme was the desire to maintain a level of resiliency despite the challenges of the work. This touched on finding an optimal balance in dealing with clients experiencing homelessness – connecting with individuals in a way that fosters trust, rapport, and an authentic alliance, but that also allows the worker to stay in control, to regulate their emotions, and remain resilient despite the challenging and sometimes upsetting material being shared.

[D]oing this job, just after a year and a half, you can see the hardness that comes over you – which is good in a way, because it gives you an ability not to be controlled at home by those thoughts and memories and what you’ve seen and what you’ve dealt with.

– Shelli

*I’m pretty good at not taking it home and not letting it really affect me personally. Sometimes that’s scary because I think, “I hope I’m still sensitive,” because you hear these horrible situations and you remember how you used to feel initially and you think, “I don’t have that feeling anymore.” So you worry that you’re changing in terms of becoming harder, but I think it’s a good thing because if I let those stories affect me personally I probably wouldn’t be here [in this role]*.

– Norma

This desire to stay strong was also linked to the obligation to do one’s best for the client, with the view that emotionally mirroring clients would not only be damaging to oneself, but importantly would not be effective in working with clients and supporting them to reach sustainable solutions to their issues and concerns.

You have to be really careful about taking on other people’s emotions because you have your own life to deal with outside of the service. So you just have to remind yourself that although this is your work, and you can be compassionate and empathize, you have to really look after yourself and have that self-care put in place. It has to be there because you will burn yourself out… And that’s not just for yourself, that’s for the client too because you’ve got to provide them the best service you can.

– Donna

…[T]hey need someone there that’s strong and that’s not going to sit there and crumble with them. I don’t think it would be good if you sat there and joined them in the sadness and pass tissues around and all that sort of stuff because, I don’t know, for me I just don’t think it’s a good look. You can have empathy and relate to them emotionally and feel it, but rise above it because they’re the ones reaching out to you to grab your hand…

– James

##### Separating work concerns and personal life

Workers also emphasized the need for clear lines between work and home life.

I go home to my family and start a new day when the key goes in the front door.

– Ed

Leave it at work. I don’t take this home with me. I’ve got a new role when I go home.

– Andy

##### Accepting the limits of what can be done

Reconciling a strong motivation to help clients versus the realities of what could be achieved was another component of the theme bounded empathy. Workers expressed a longing to provide a panacea to help all their clients to overcome their hardships – however, this was tempered with the clear pragmatic recognition that many clients experience complex problems and setbacks, and that often small incremental change was all that might be achieved.

In the early days I wanted to save everyone… I’ve realized I can’t save everyone.

– Audrey

It’s their journey, their stuff. I’m only there in a very small role, but a very big role, to facilitate what it is they need to do on their journey to reach their desired outcome.

…

I can’t want their success more than they want it, and I can’t set their goals because they’re probably unrealistic and unattainable… My role is to walk with their permission, their journey, but alongside, and encourage them and help them to stay on track to their case plans, to their life’s goals.

– Maddie

Accepting the boundaries of one’s own personal sphere of influence, for instance by deferring to a higher power or religion, was another way workers reported dealing with and working through challenges in the face of seeming futility or personal ineffectiveness.

…[P]raying is big. So at the end of the day I say Lord here, they’re yours, you love them as much as you love me, and I don’t know what I can do…[I] leave it at the foot of the cross and go okay fine, I’ve done my job, and I’m hurt, but I don’t want to carry it on, so help me.

– Nadine

## Discussion

The present work examined how frontline workers in the homelessness sector deal with the suffering of their clients. These workers perform their duties against a difficult backdrop: a complex client base, ongoing themes of crisis, and distress, plus the stigma of their profession and minimal recompense for ‘dirty work’ (Hughes, 1958; Baker et al., 2007; Chigavazira et al., 2013). These factors combine to create an environment in which workers are vulnerable to workplace stress and burnout (Maslach, 2003). We examined two ways workers could deal with these demands and still function in their role: through organizational identification, and by creating emotional distance from clients through infrahumanization. We considered whether organizational identification might provide workers with the social capital they need to thrive in their roles. We also tested whether workers who infrahumanized clients might be less vulnerable to the negative effects of being exposed to their suffering (Vaes and Muratore, 2013; Trifiletti et al., 2014).

### Clients’ Suffering and the Florence Nightingale Effect

When looking at perceived client suffering, we found evidence of a mediating role for organizational identification in two key relationships. Perceived suffering positively predicted job satisfaction, and the direct effect of suffering on satisfaction was no longer significant once the indirect path via identification was accounted for. Similarly, perceived client suffering predicted less burnout, and this again was fully mediated by organizational identification. These mediation models provide preliminary evidence to indicate that acknowledging client suffering may increase job satisfaction and reduce burnout by galvanizing organizational identification. We introduce this novel finding as the ‘Florence Nightingale effect’.

These results provide a counterpoint to the literature on vicarious exposure to the suffering of others in medical settings (Vaes and Muratore, 2013; Trifiletti et al., 2014). Indeed this literature predicts that the practice of recognizing clients’ suffering would take a toll on workers and lead to higher burnout and less satisfaction. There is a large literature pointing to the deleterious effects of vicarious trauma for workers in the human services (Miller et al., 1995; Maslach et al., 2001; Baker et al., 2007; Gleichgerrcht and Decety, 2013). However, this was not the case in the present study. Instead, we see in the present research that recognizing suffering is predictive of positive workplace outcomes – *through* identification with the organization.

The Florence Nightingale effect therefore represents a novel contribution to the literature as a new approach to understanding the role of recognizing suffering for positive occupational identities. It contributes to the literature on organizational identification and ‘dirty work’, which highlights the value of identity solidarity in stigmatized occupations (Ashforth and Kreiner, 1999). The homelessness sector is ostensibly neither high-status nor well-remunerated – but here, suffering may be the ingredient that adds status or moral value to this occupational identity. If relieving human suffering is the *raison d’être* for the organization and its efforts, then recognizing that suffering in others conceivably provides an avenue for reinforcement of a meaningful organizational identity, and in turn for the concomitant benefits of higher job satisfaction and less burnout. Concepts of futility, inefficacy, or fatalism in the face of insurmountable human need might be better thwarted together than alone – as ‘groups often can sustain beliefs that individuals cannot’ (Ashforth and Kreiner, 1999, p. 421). Perhaps in this way, others’ suffering can be seen as a call to arms and a motivating force, rather than a dispiriting realization of the human condition. We thereby draw on the social identity literature (Haslam et al., 2004, 2009; Riketta and Dick, 2005; van Dick and Haslam, 2012) and introduce a different theoretical perspective on how workers might deal with the suffering of their clients.

### Protective Infrahumanization

Drawn from a homelessness services context, our data did not reveal a negative association between infrahumanization and burnout, even for workers with high client contact. We were unable to explain patterns of burnout or job satisfaction in this cohort by reference to infrahumanization. This is in contrast to the findings reported by Vaes and Muratore (2013), in which medical workers who infrahumanized patients reported less burnout, particularly for those working in high contact roles. These findings also diverge from those indicated by Trifiletti et al. (2014), who found a link between patient infrahumanization and lower stress symptoms for nursing staff with high affective commitment to the organization and patients.

There is the possibility that workers might be engaging in infrahumanization as a normative practice. Interestingly, qualitative evidence showed that nearly two–thirds of workers overtly discussed their connection with clients in terms of bounded empathy – where authentic connection to and understanding of clients’ circumstances is paramount, but the empathetic connection has strict boundaries, and suffering is “left at the door” when workers go home. This offers an interesting insight into how workers are conceptualizing their clients’ suffering and creating functional distance. More specifically, the qualitative findings shed light on the way suffering can be conceptualized by workers, and how workers articulate the role of empathy in their practice (for instance, being motivated by social justice ideals, and balancing client need with the need for workers to set boundaries; Gerdes and Segal, 2009).

Another explanation for why we did not find protective infrahumanization relates to the differences between social pain and physical pain. Our mediation models indicate that perceiving others’ social pain and suffering may actually serve to *bolster* workplace functioning via organizational identification. This is markedly different to the extant literature on exposure to others’ physical pain, where emotional distance away from such exposure buffered against burnout. We thereby add to an emerging literature on critical differences in the psychology of social versus physical pain (Iannetti et al., 2013; Woo et al., 2014). Indeed, while there are commonalities between the hurtful experiences of social pain (such as social exclusion or ostracism) and physical pain (MacDonald and Leary, 2005), there are key points of difference between these two pains. For instance, enduring and prevailing through physical pain can be seen by others as a sign of strength or moral virtue (Bastian et al., 2014b), whereas social pain may be seen as detractive, perhaps signaling reduced social standing, or as reliably giving rise to negative affect and lowered self-esteem (Smart Richman and Leary, 2009). This suggests that the psychological corollaries of exposure to others undergoing such pains might be quite different, because the meaning, social functions, and value of those pains are different. Accordingly, we could expect to see differing patterns in how people respond to that exposure, consistent with the findings of this study.

### Limitations and Future Research

This study has certain limitations. Despite the advantages of a field sample over a convenience sample in terms of ecological validity, we note the need for further research to rule out whether distinctive features characterizing this sample’s organization are borne out in other organizations within and beyond a homelessness context. For instance, it would be of interest to examine whether the Florence Nightingale effect prevails in other ‘helping’ professions, and organizational settings where there are ostensibly different relationships between the worker and care-recipient, as well as different organizational goals and norms. Such contexts might include clinical psychology practice, or delivery of non-medical humanitarian aid (e.g., civil capacity-building) by military and non-government organizations. Similarly, while we focused on organizational identification, future research might want to focus on examining whether similar effects are obtained when professional identification is measured. Strong professional identity could also feasibly serve a protective function. It would also be valuable to quantitatively examine the role of perceived efficacy to ameliorate suffering. For homelessness workers, recognizing social pain in their clients may be associated with positive functioning because it is considered within their collective ability to alleviate that suffering. Specifically probing these and other themes (such as interpersonal authenticity and perceived efficacy to ameliorate different kinds of suffering) could deliver further insights into how workers might be framing these challenges.

Further, sampling those presently employed may have inadvertently excluded workers who are struggling or already burnt out, with attrition of these workers from the sector making their views and experiences harder to access. While the sample size of the present study was adequate, it was also smaller than the other studies in the literature. We took steps to mitigate this by utilizing bootstrapping in our analyses, with the aim of increasing power and coverage probability (Fritz and MacKinnon, 2007), and gathered valuable qualitative information for analysis. However, future research will valuably add to the literature by enlisting larger sample sizes, diversifying how relevant constructs are measured in an effort to avoid common method variance (Antonakis et al., 2010, 2014); and should canvass the experiences of former workers in addition to current employees. It would also be important to consider the pathways for workers who *do* experience burnout as a subset – what factors might predispose workers, and are there boundary conditions to the ostensibly protective value of recognizing suffering collectively.

Assessing how workers attribute emotions with vignettes of course only approximates the process of considering a real client’s emotional state. However, it allowed us to respect client confidentiality constraints, and to target emotion attribution for individuals, not clients generally. We have also argued that the use of emotion attributions rather than traits to measure infrahumanization is a more direct way to target emotional distancing practices. This differs from the approach taken by Trifiletti et al. (2014), who examined patient infrahumanization in terms of the attribution of traits rather than emotions. Their infrahumanization measure involved ratings on a smaller set of four uniquely human and four non-uniquely human traits validated for an Italian cohort. This differs slightly from other trait attribution studies in the literature (e.g., Andrighetto et al., 2014) in that eight rather than 14 traits were tested – but more relevantly, our study focused on emotions, and only along the uniquely human dimension (based on the methodology of Vaes and Muratore, 2013). This difference alone should not explain why protective infrahumanization was not supported in the present dataset, given that Vaes and Muratore (2013) also deployed these same measures. Nevertheless, future studies could confirm whether and when these subtle differences in the measurement of humanness are important.

In terms of mediation, we have found evidence of a mediating role for organizational identification in explaining the respective relationships between perceived suffering and reduced burnout and increased job satisfaction. We tested reverse models with suffering as the outcome variable, and the data do not support these reverse models. Furthermore, as discussed, qualitative data yielded nominal support for our posited mediation models. Nevertheless, due to the cross-sectional design we used in this study, we cannot rule out the possibility that an externality or unmeasured variable or variables might provide an alternative explanation for these findings (Hayes, 2013). Experimental studies would valuably contribute to this evidence base by providing data that could facilitate the drawing of causal inferences (Antonakis et al., 2010, 2014). There is also a broader need to augment existing literature on protective infrahumanization with experimental studies, with recent research emerging in response to this need (see for example, Cameron et al., 2015). In sum, while our findings deviate from prior literature, and this can be approached and understood in several ways, we suggest there are sound theoretical reasons for why our results differ, as canvassed above.

## Conclusion

It is a special undertaking to respond to the suffering of others and support those in need, and doing so carries both reward and challenges. Frontline workers in the homelessness sector routinely deal with clients who are suffering, and this challenging environment means they are vulnerable to compassion fatigue and burnout. Previous research suggested that infrahumanization of patients and clients could be protective for workers in a medical context. However, we failed to find evidence that infrahumanization explained workplace functioning in the form of burnout and satisfaction. Rather, with two mediation models we report that perceived client suffering predicts reduced burnout and increased satisfaction, with a mediating role for organizational identification in each of these relationships. We present this as the Florence Nightingale effect – whereby perceived client suffering is linked to increased identification with the organization, which in turn predicts less burnout, and more job satisfaction. Ultimately, seeing another human being suffering is part of the everyday experience for workers in the homelessness sector, and social psychological perspectives have much to offer in extending our understanding of the difficulties facing workers in the sector. In the meantime, people experiencing homelessness rely on the support and generosity of these workers: their important work continues.

## Conflict of Interest Statement

The authors declare that the research was conducted in the absence of any commercial or financial relationships that could be construed as a potential conflict of interest.

## Footnotes

1 As anticipated, we surmise that the latter reflects the somewhat gender-segregated nature of service delivery, where participants who frequently work with clients of one gender are less likely to work often with clients of the other.

2 We detected significant differences between vignettes in negative secondary and primary emotions, with participants attributing ‘Denise’ a greater median number of negative secondary (*Mdn* = 4.00) and negative primary (*Mdn* = 5.00) emotions compared to ‘Warren’ (secondary: *Mdn* = 3.00, *T =* 813.00, *p* = 0.007, *r* = 0.25; primary: *Mdn* = 4.00, *T =* 893.50, *p* < 0.001, *r* = 0.32). All other comparisons between vignettes were non-significant. Differences in emotion attribution between vignettes were tentatively anticipated, given prior research indicating differential infrahumanization of men and women, and criminals (see Hetey and Eberhardt, 2014, for review). However, we were satisfied that key variables were highly correlated between vignettes, and that having more than one vignette reduced the risk that any observed effects would be attributable to an artifact of the materials.

3 Reliability for two-item scales is a Spearman-Brown-corrected correlation (Eisinga et al., 2013).

4 Variables were screened for violations of normality assumptions. Client suffering and job satisfaction were negatively skewed (suffering: -1.34, *SE* = 0.306; job satisfaction: -1.64, *SE* = 0.309), which was resolved by square root and log10 transformation, respectively, (suffering = -0.77, *SE* = 0.306; job satisfaction = -0.58, *SE* = 0.309). Untransformed data were used for bootstrapped mediation analyses (Hayes, 2013; see further at Footnote 5 below).

5 We conducted mediation analyses with untransformed data, then repeated mediation analyses with transformed data; the indirect effect of suffering via workplace identification on each of the workplace functioning variables prevailed regardless of whether transformed or untransformed data were used. Therefore the untransformed data are presented, consistent with convention (Hayes, 2013).

6 Names have been substituted to protect participants’ confidentiality.
